# A comparison of active versus passive methods of responding to rapid diagnostic blood culture results

**DOI:** 10.1017/ash.2022.26

**Published:** 2022-05-02

**Authors:** Elisabeth L. Chandler, Katie L. Wallace, Elizabeth Palavecino, James R. Beardsley, James W. Johnson, Vera Luther, Christopher Ohl, John C. Williamson

**Affiliations:** 1 Department of Pharmacy, Lee Health, Fort Myers, Florida; 2 Department of Pharmacy, University of Kentucky HealthCare, Lexington, Kentucky; 3 Department of Pathology, Wake Forest School of Medicine, Winston Salem, North Carolina; 4 Department of Pharmacy, Atrium Health–Wake Forest Baptist, Winston Salem, North Carolina; 5 Section on Infectious Diseases, Department of Internal Medicine, Wake Forest School of Medicine, Winston Salem, North Carolina

## Abstract

**Objective:**

To compare 2 methods of communicating polymerase chain reaction (PCR) blood-culture results: active approach utilizing on-call personnel versus passive approach utilizing notifications in the electronic health record (EHR).

**Design:**

Retrospective observational study.

**Setting:**

A tertiary-care academic medical center.

**Patients:**

Adult patients hospitalized with ≥1 positive blood culture containing a gram-positive organism identified by PCR between October 2014 and January 2018.

**Methods:**

The standard protocol for reporting PCR results at baseline included a laboratory technician calling the patient’s nurse, who would report the critical result to the medical provider. The active intervention group consisted of an on-call pager system utilizing trained pharmacy residents, whereas the passive intervention group combined standard protocol with real-time in-basket notifications to pharmacists in the EHR.

**Results:**

Of 209 patients, 105, 61, and 43 patients were in the control, active, and passive groups, respectively. Median time to optimal therapy was shorter in the active group compared to the passive group and control (23.4 hours vs 42.2 hours vs 45.9 hours, respectively; P = .028). De-escalation occurred 12 hours sooner in the active group. In the contaminant group, empiric antibiotics were discontinued faster in the active group (0 hours) than in the control group and the passive group (17.7 vs 7.2 hours; P = .007). Time to active therapy and days of therapy were similar.

**Conclusions:**

A passive, electronic method of reporting PCR results to pharmacists was not as effective in optimizing stewardship metrics as an active, real-time method utilizing pharmacy residents. Further studies are needed to determine the optimal method of communicating time-sensitive information.

Delay in appropriate antimicrobial therapy leads to increased risk of mortality and adverse patient outcomes.^1–3^ Rapid diagnostics provide faster time to pathogen identification and can help decrease broad-spectrum antibiotic use, improve time to optimal antibiotics, and decrease healthcare expenditures.^4–6^ The ability to optimize stewardship efforts and patient outcomes depends upon having personnel available to respond to test results. Although the largest benefit has been demonstrated when rapid diagnostic testing is combined with active antimicrobial stewardship interventions,^7–9^ the best method of communicating rapid diagnostic results remains unknown. However, competing obligations or financial barriers may result in limited availability of infectious disease or stewardship-trained staff, especially on evenings and weekends.

Technological advances allow decision support and communication tools to be integrated into the electronic health record (EHR). In healthcare settings with limited availability of stewardship-trained staff, automated reporting of rapid diagnostic results within the EHR to clinical pharmacists has the potential to produce comparable stewardship outcomes. The benefit of this passive method of communicating rapid diagnostic results compared to active response by dedicated staff is unclear.

Rapid diagnostic technology in the form of multiplex polymerase chain reaction (PCR) helps decrease time to organism identification and identifies genetic elements that confer resistance. At the time of this study, Atrium Health Wake Forest Baptist (AHWFB) used this technology for blood cultures of patients bedded in an intensive care unit (ICU) or an oncology unit. These PCR results allow clinicians to transition from empiric, broad-spectrum antibiotic therapy to optimal, definitive therapy more quickly.

We compared 2 methods of communicating rapid diagnostic PCR results of blood cultures with a gram-positive bacteria to pharmacists: (1) an on-call pager system utilizing dedicated pharmacy residents to report results along with antibiotic recommendations to the medical provider and (2) a telephone call from the lab to the patient’s nurse to report results combined with real-time EHR in-basket notifications to clinical pharmacists.

## Methods

This retrospective, observational, single-center study was conducted at AHWFB, an 885-bed academic medical center. Throughout the study period, blood culture samples were analyzed using rapid diagnostic technology but only for patients bedded in an ICU or an oncology unit. Inoculated blood-culture bottles were first incubated using the BD BactecFX blood culture instrument (Becton Dickinson, Franklin Lakes, NJ). When the blood-culture bottle was flagged as positive, a multiplex PCR assay (BioFire FilmArray Blood Culture Identification Panel, Biofire, Salt Lake City, UT) was used for organism identification and to identify certain genes that encode for resistance (eg, *mecA* and *vanA*, which confer resistance to oxacillin for staphylococci and vancomycin for enterococci, respectively). After growth on solid media, organism identification was confirmed using matrix-assisted laser desorption/ionization-time of flight (MALDI-TOF) technology. For study purposes, only cultures that yielded a gram-positive bacteria were assessed. The study was approved by the AHWFB Institutional Review Board.

This study comprised 3 study periods, 1 of which was a control period when rapid diagnostic testing was being performed as described above and multiplex PCR results were reported by telephone from the laboratory technologist to the patient’s nurse 24 hours a day, 7 days a week. Because positive blood cultures were considered a critical laboratory result, the nurse was obligated to inform the medical provider immediately according to hospital policy.

In the second study period, the laboratory technologist paged an on-call pharmacy resident to report the PCR result. The nurse was not notified of results during this period. The pharmacy resident was responsible to inform the medical provider directly and to provide initial recommendations about antimicrobial therapy. Before the start of this period, the pharmacy residents received dedicated instruction on antimicrobial stewardship principles and optimal antibiotic regimens for specific pathogens. This approach was considered the “active” method of responding to results and was in operation 24 hours a day, 7 days a week. Pharmacy residents participating in the active method were postgraduate year 1 (PGY1) pharmacy residents and postgraduate year 2 (PGY2) specialty residents. The PGY2 residents were available as back-up to the PGY1 residents in case of questions. All recommendations were documented by the PGY1 residents and were verified for appropriateness within 24 hours by a PGY2 resident. Pharmacists trained in infectious diseases were also available to the PGY2 residents for consultation.

In the third study period, PCR results were reported to the patient’s nurse by the laboratory technologist and subsequently by the nurse to the medical provider, as was done in the control group. However, results were also reported in real time as in-basket messages within the EHR (Epic Systems, Verona WI) to clinical pharmacists responsible for the patient’s care. Pharmacist dashboards and in-baskets were filtered by “pools” that corresponded to their specific service or nursing unit assignment. New messages displayed in bold font in the in-basket, but the receiving pharmacist had to notice the appearance of a new message and click into the message to see the culture information. This approach was considered the “passive” method of responding to results. Although clinical pharmacists at AHWFB provide antibiotic recommendations routinely during patient care, the passive method of reporting had no predefined requirement for making antibiotic recommendations. Prior to implementing in-basket messaging, pharmacists received e-mail communication with a description of how to use the tool and its purpose, but additional education about antimicrobial stewardship principles or pathogen-specific antibiotic regimens was not provided. Even though in-basket results were reported real time, a dedicated clinical pharmacist was not always available (eg, during evening, weekend, or overnight hours) to act on the results.

Patients included in this study met the following criteria: aged ≥18 years, admitted to an ICU or oncology unit, and ≥1 positive blood culture containing a gram-positive organism identified by PCR. We excluded patients with polymicrobial infection, concomitant infection caused by a different organism that prevented change to optimal antibiotic therapy, antibiotics started for a positive blood culture before admission to AHWFB, or death prior to organism identification.

Patients were identified from a computer-generated report of positive blood-culture PCR results. Data were collected retrospectively via chart review of the EHR. The study was performed between October 2014 and January 2018. Data were collected during a 9-month period for the control group and separate 4-month periods for the 2 intervention groups. We included a transition period between the active and passive groups to implement in-basket messaging and reverse the active process.

The primary outcome measure was time from blood culture collection to first dose of optimal antibiotic therapy. Optimal therapy was defined as an evidence-based antibiotic regimen supported by reputable therapeutic guidelines, microbiology results, patient-specific considerations, and documented rationale. Secondary outcomes included days of antibiotic therapy (DOT), time to de-escalation, time to microbiologically active therapy, length of hospital stay from time of positive culture, and inpatient mortality. DOT was determined by the number of calendar days that the patient received at least 1 dose of an antibiotic. DOT was calculated for antibiotic received, and DOTs were summed for comparison purposes. De-escalation was defined as discontinuation of 1 or more antibiotics or converting from a broad-spectrum to narrow-spectrum agent. Time to de-escalation was analyzed only for patients whose initial antibiotic was not optimal. Microbiologically active therapy was defined as an antibiotic to which the pathogen was proven susceptible.

For analysis purposes, patients were further categorized as having contaminants or pathogens by the investigators. Criteria for contaminant or pathogen classification included clinical suspicion of infection per documentation in the EHR, organism identified, number of positive cultures, site of blood draw, and time to positivity of culture result. This determination may have occurred in real time by the pharmacy resident during the active method of reporting.

We used the χ^2^ test or the Fisher exact test to analyze categorical data. One-way analysis of variance was used for ordinal and continuous data that were normally distributed. The Kruskal-Wallis test was used for ordinal and continuous data that were not normally distributed.

## Results

**Fig. 1.**
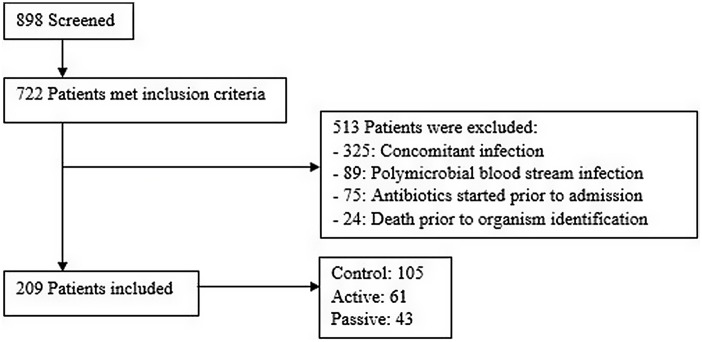
Patient screening.

Of 722 adult patients with a gram-positive organism identified by PCR, 513 patients were excluded. The most common reason for exclusion was concomitant infection caused by a different organism that prevented optimal antibiotic therapy (n = 325). For instance, a patient with concomitant pneumonia or urinary tract infection may require broad-spectrum antibiotics to cover the bloodstream infection in addition to the primary infection. Other reasons for exclusion are listed in Figure 1. Moreover, 209 patients met study criteria with 105 patients in the control group, 61 patients in the active group, and 43 patients in the passive group (Fig. 1). We identified 2 notable differences in patient characteristics: more patients in the passive group were located in an ICU and more patients in the active group had a hematological malignancy (Table 1). The average age of patients in the study population was 60 years; 53% were male; and 74% were in an ICU. In the patients with noncontaminant bacteremia, the median Pitt bacteremia score was 2 and the median number of systemic inflammatory response syndrome (SIRS) criteria met was 3 (Table 1).

**Table 1.** Patient Characteristics

Characteristic	Control (n = 105)	Active (n = 61)	Passive (n = 43)	*P* Value
Age, mean y ± SD	61±16	58±15	62±17	NS
Male gender, no. (%)	63 (60)	27 (44)	21 (49)	NS
ICU Location, no. (%)	80 (76)	38 (62)	36 (84)	.036
Oncology service, no. (%)	20 (31)	15 (42)	12 (38)	NS
Hematologic malignancy, no. (%)^a^	10 (9)	14 (22)	2 (4)	.009
Charlson comorbidity index score, median	4	5	5	NS
Pitt bacteremia score, median^b^	1	2	2	NS
SIRS criteria, median^b^	3	3	3	NS

Note. SD, standard deviation; ICU, intensive care unit; SIRS, systemic inflammatory response syndrome.

a Leukemia or lymphoma.

b Not reported for patients with contaminants.

Of the bacteria isolated, the most prevalent species were coagulase-negative staphylococci (45.5%) and *Staphylococcus aureus* (32.5%) (Table 2). The *mecA* gene, which confers oxacillin resistance, was detected by PCR for 55.9% of *S. aureus* isolates. Of the 209 cultured organisms, 76 (36.4%) were considered contaminants, and 74 (97.4%) of these were coagulase-negative staphylococci. Only 5 (2.4%) vancomycin-resistant *Enterococcus* (VRE) isolates were detected.

**Table 2.** Isolated Organisms

Study Group	Control (n = 105)^a^	Active (n = 61)^a^	Passive (n = 43)^a^	Total (n = 209)^a^
Oxacillin-resistant *Staphylococcus aureus*	23 (21.9)	8 (13.1)	7 (16.3)	38 (18.2)
Oxacillin-susceptible *Staphylococcus aureus*	13 (12.4)	8 (13.1)	9 (20.9)	30 (14.4)
Oxacillin-resistant coagulase-negative staphylococci	30 (28.6)	18 (29.5)	12 (27.9)	60 (28.7)
Oxacillin-susceptible coagulase-negative staphylococci	18 (17.1)	12 (19.7)	5 (11.6)	35 (16.7)
*Streptococcus* spp	15 (14.3)	10 (16.4)	7 (16.3)	32 (15.3)
Vancomycin-susceptible *Enterococcus* spp	5 (4.8)	2 (3.3)	2 (4.7)	9 (4.3)
Vancomycin-resistant *Enterococcus* spp	1 (1.0)	3 (4.9)	1 (2.3)	5 (2.4)

a Values expressed as no. (% of group).

Outcome measures for patients with noncontaminant bacteremia are presented in Table 3. Median time to optimal therapy (primary outcome) was shorter in the active group (23.4 hours), compared to the control group and the passive group, respectively (45.9 and 42.2 hours; *P* = .028). Initial antibiotic therapy was deemed optimal for 15 patients (14.3%) in the control group, 13 patients (21.3%) in the active group, and 4 patients (9.3%) in the passive group. Among patients whose antimicrobial therapy was de-escalated, the median time to de-escalation was ∼12 hours shorter in the active group than in the other groups [46.6 hours (control) vs 34.4 hours (active) vs 46.5 hours (passive)], but this did not reach statistical significance (*P* = .23). Of the candidates for de-escalation, 45 (90%) of 50 patients in the control group were de-escalated, 23 (100%) of 23 patients in the active group were de-escalated, and 25 (89.3%) of 28 patients in the passive group were de-escalated (*P* = .306). No statistical difference was detected in time to microbiologically active therapy or DOT. In-hospital mortality was 23% in the control group, 17% in the active group, and 25% in the passive group (*P* = .667). Length of hospital stay after positive culture was shorter in the passive group (7.9 days) compared with the control (10.2 days) and active (14.0 days) groups (*P* = .019). Only 1 episode of hospital-onset *C. difficile* infection occurred in the study population.

**Table 3.** Outcomes for Patients with Noncontaminant Bacteremia

Outcome	Control (n = 65)	Active (n = 36)	Passive (n = 32)	*P* Value
Time to optimal therapy, median h	45.9	23.4	42.2	.028^a^
Time to de-escalation, median h	46.6	34.4	46.5	.228
Time to microbiologically active therapy, median h	1.6	2.0	2.8	.789
Days of therapy, median	14.0	15.5	11.0	.265
In-hospital mortality, no. (%)	15 (23)	6 (17)	8 (25)	.667
Length of stay from positive culture, days	10.2	14.0	7.9	.019^b^

a *P* = .011 active vs control; *P* = .164 active vs passive.

b *P* = .017 active vs control; *P* = .102 active vs passive.

Among patients whose cultures represented contaminants, antibiotic discontinuation occurred in a timely fashion during the active method (Table 4). Patients with contaminants in the active group had shorter antibiotic durations and fewer DOT. In the active group, there was minimal continuation of antibiotics after the PCR result. Only 1 patient in the active group whose culture was considered a contaminant received an antibiotic ≥24 hours after the PCR result. Also, 28 patients with contaminants never received an antibiotic. Of those who received empiric therapy, the median duration was significantly shorter in the active group: 17.7 hours (control group) versus 0 hours (active group) versus 7.2 hours (passive group) (*P* = .007).

**Table 4.** Antibiotic Durations among Patients with Contaminants

Variable	Control (n=40)	Active (n=25)	Passive (n=11)	*P* Value
Total antibiotic duration, median h	13.0	1.9	4.8	.961
Antibiotic duration after PCR result,^a^ median h^b^	17.7	0	7.2	.007
Days of therapy, median	2	1	2	.643
Received ≥24 h of antibiotic after PCR result, no. (%)	11 (28)	1 (4)	2 (18)	.059

Note. PCR, polymerase chain reaction.

a Results reported only for patients who received antibiotics (n = 24, 15, and 6, respectively)

b Zero values given for patients whose antibiotic was discontinued before PCR result.

## Discussion

Rapid diagnostic tests in combination with antimicrobial stewardship have been shown to decrease time to optimal therapy and to assist with earlier de-escalations than rapid diagnostics alone.^10,11^ A meta-analysis demonstrated decreased mortality in patients with bloodstream infection when antimicrobial stewardship efforts were combined with rapid diagnostics, but this difference was not seen with rapid diagnostics alone.^12^ However, stewardship initiatives differ in processes between institutions, and the best practice for reporting and responding to positive culture results has yet to be determined.^13–15^ Furthermore, familiarity with rapid diagnostic technologies and level of formal infectious diseases or antimicrobial stewardship training may have differed among staff and may have affected their capability to interpret and respond to rapid diagnostic results.^16^ This study is unique in that 2 different methods of communication of rapid diagnostic results, an active and a passive method, were compared to rapid diagnostics alone.

With advancing technology, health systems are seeking to capitalize upon tools in the EHR. Because a 24-hour-per-day, 7-day-per-week process involving active response to positive culture results could not be sustained by dedicated staff at our institution, a strategy to use in-basket notifications within the EHR was implemented. We sought to determine whether this automated but passive process was as effective as the active process in optimizing antimicrobial stewardship metrics.

The results of this study generally favor the active process that included a dedicated pharmacist who provided advice about antibiotic therapy at the time the positive culture was reported. Measures reflecting the ability to make timely antibiotic interventions were best in the active group, including time to optimal therapy and duration of antibiotics among patients with contaminants. The active group also had the lowest rate of in-hospital mortality. Although this is a compelling result, the sample size was too small to make conclusions about the impact of the active method on mortality compared to other methods.

In this study, we evaluated only pharmacists in the intervention groups; however, the pharmacists that participated in the active group were enrolled in a pharmacy residency training program. These residents received targeted training to participate in the study, but none of them had completed specialty training programs in infectious diseases or antimicrobial stewardship. As such, it would seem feasible to utilize pharmacy residents in responding to positive blood-culture results. However, for a large medical center like AHWFB, a robust number of personnel would be required to make this model sustainable in the long term. Revising the model so that it does not operate 24 hours a day, for instance, may improve the feasibility of using pharmacy residents, but that may come with some sacrifice in outcomes achieved. Whether these results could be extrapolated to other trainees remains unclear. Notably, investigators have obtained conflicting results when comparing infectious disease fellows and pharmacists with regard to antibiotic audit and review activities.^17,18^ Processes that involve messaging medical providers should be considered in future studies.

This study had several important limitations. The retrospective nature of the study limited the accuracy of the data collected. Inclusion criteria as well as several clinical outcome measures were dependent upon appropriate documentation in the EHR, and the analysis of this information by study investigators has the potential for misinterpretation (eg, pathogen vs contaminant).

An inherent limitation of in-basket reporting is that response to these notifications was limited to the working hours of the clinical pharmacists, which was generally daytime hours. In-basket messages of positive cultures that appeared after daytime working hours may have been unread until the following morning. The study investigators did not assess the time between in-basket notification and when it was read by the pharmacist. Additionally, the study did not assess how often the clinical pharmacists made an antibiotic recommendation in response to the in-basket notifications.

In this study, we evaluated 2 different methods of communication, as well as differences in the pharmacists’ level of knowledge about rapid diagnostic tests. An important distinction between the active and passive methods is that while the in-basket tool was made available to clinical pharmacists, there was no additional stewardship training provided to them. It is difficult to determine whether the greatest impact was wholly due to the method of communication or whether the level of instruction and familiarity of rapid diagnostic tests played a role in impacting the results. The pharmacy residents participating in the active method received instructions and education about rapid diagnostic tests and interpretation. Although this was not considered formal training, it may have afforded them greater familiarity compared to pharmacists with the passive method. This aspect should be considered when interpreting the results.

Several differences among the study groups were noted, such as the proportion located in an ICU or with hematologic malignancy, which has the potential to affect outcomes. The greater number of patients with hematologic malignancy in the active group was likely to have contributed to a longer hospital length of stay and DOT for these patients. Additionally, the hospital length of stay and DOT may have been influenced (shortened) by a new outpatient parenteral antibiotic therapy service that was implemented during the period of passive reporting. This service aimed to discharge patients who would have otherwise had to stay in the hospital to complete their entire course of antibiotics, eg, patients with infections related to substance use disorder. Lastly, the sample size was relatively small, particularly for the intervention groups, so the study may have been underpowered to detect a difference for certain measures.

In conclusion, antibiotic utilization associated with passive reporting of rapid diagnostic blood culture results using automated in-basket notifications to pharmacists was not equivalent to an active process with dedicated and trained staff. Additional investigations are warranted to determine the most effective method of responding to rapid diagnostic blood-culture results. These data help to solidify the role of dedicated, stewardship-trained staff in maximizing stewardship benefits.

## Supplementary material

For supplementary material accompanying this paper visit https://doi.org/10.1017/ash.2022.26. click here to view supplementary material

## References

[1] Liu VX , Fielding-Singh V , Greene JD , et al. The timing of early antibiotics and hospital mortality in sepsis. Am J Respir Crit Care Med 2017;196:856–863. 28345952 10.1164/rccm.201609-1848OC PMC5649973

[2] Kang CI , Kim SH , Park WB , et al. Bloodstream infections caused by antibiotic-resistant gram-negative bacilli: risk factors for mortality and impact of inappropriate initial antimicrobial therapy on outcome. Antimicrob Agents Chemother 2005;49:760–766. 15673761 10.1128/AAC.49.2.760-766.2005 PMC547233

[3] Lodise TP , Mckinnon PS , Swiderski L , Rybak MJ. Outcomes analysis of delayed antibiotic treatment for hospital-acquired *Staphylococcus aureus* bacteremia. Clin Infect Dis 2003;36:1418–1423. 12766837 10.1086/375057

[4] Perez KK , Olsen RJ , Musick WL , et al. Integrating rapid pathogen identification and antimicrobial stewardship significantly decreases hospital costs. Arch Pathol Lab Med 2013;137:1247–1254. 23216247 10.5858/arpa.2012-0651-OA

[5] Beuving J , Wolffs PF , Hansen WL , et al. Impact of same-day antibiotic susceptibility testing on time to appropriate antibiotic treatment of patients with bacteraemia: a randomised controlled trial. Eur J Clin Microbiol Infect Dis 2015;34:831–838. 25527447 10.1007/s10096-014-2299-0

[6] Banerjee R , Teng CB , Cunningham SA , et al. Randomized trial of rapid multiplex polymerase chain reaction-based blood culture identification and susceptibility testing. Clin Infect Dis 2015;61:1071–1080. 26197846 10.1093/cid/civ447 PMC4560903

[7] Huang AM , Newton D , Kunapuli A , et al. Impact of rapid organism identification via matrix-assisted laser desorption/ionization time-of-flight combined with antimicrobial stewardship team intervention in adult patients with bacteremia and candidemia. Clin Infect Dis 2013;57:1237–1245. 23899684 10.1093/cid/cit498

[8] Porter AM , Bland CM , Young HN , et al. Comparison of pharmacist-directed management of multiplex PCR blood culture results with conventional microbiology methods on effective and optimal therapy within a community hospital. Antimicrob Agents Chemother 2019;63:e01575–18. 30323051 10.1128/AAC.01575-18 PMC6325225

[9] Neuner EA , Pallotta AM , Lam SW , et al. Experience with rapid microarray-based diagnostic technology and antimicrobial stewardship for patients with gram-positive bacteremia. Infect Control Hosp Epidemiol 2016;37:1361–1366. 27767002 10.1017/ice.2016.175

[10] Claeys KC , Heil EL , Hitchcock S , Johnson JK , Leekha S. Management of gram-negative bloodstream infections in the era of rapid diagnostic testing: impact with and without antibiotic stewardship. Open Forum Infect Dis 2020;7:ofaa427. 33134414 10.1093/ofid/ofaa427 PMC7585329

[11] Bookstaver PB , Nimmich EB , Smith TJ , et al. Cumulative effect of an antimicrobial stewardship and rapid diagnostic testing bundle on early streamlining of antimicrobial therapy in gram-negative bloodstream infections. Antimicrob Agents Chemother 2017;61:e00189–17. 28630187 10.1128/AAC.00189-17 PMC5571292

[12] Timbrook TT , Morton JB , McConeghy KW , Caffrey AR , Mylonakis E , LaPlante KL. The effect of molecular rapid diagnostic testing on clinical outcomes in bloodstream infections: a systematic review and meta-analysis. Clin Infect Dis 2017;64:15–123. 27678085 10.1093/cid/ciw649

[13] Porter AM , Bland CM , Young HN , et al. Comparison of pharmacist-directed management of multiplex pcr blood culture results with conventional microbiology methods on effective and optimal therapy within a community hospital. Antimicrob Agents Chemother 2019;63:e01575–18. 30323051 10.1128/AAC.01575-18 PMC6325225

[14] Mahrous AJ , Thabit AK , Elarabi S , Fleisher J. Clinical impact of pharmacist-directed antimicrobial stewardship guidance following blood culture rapid diagnostic testing. J Hosp Infect 2020;106:436–446. 32927014 10.1016/j.jhin.2020.09.010

[15] Huang AM , Newton D , Kunapuli A , et al. Impact of rapid organism identification via matrix-assisted laser desorption/ionization time-of-flight combined with antimicrobial stewardship team intervention in adult patients with bacteremia and candidemia. Clin Infect Dis 2013;57:1237–1245. 23899684 10.1093/cid/cit498

[16] Foster RA , Kuper K , Lu ZK , Bookstaver PB , Bland CM , Mahoney MV. Pharmacists’ familiarity with and institutional utilization of rapid diagnostic technologies for antimicrobial stewardship. Infect Control Hosp Epidemiol 2017;38:863–866. 28490386 10.1017/ice.2017.67

[17] Rattanaumpawan P , Upapan P , Thamlikitkul V. A noninferiority cluster-randomized controlled trial on antibiotic postprescription review and authorization by trained general pharmacists and infectious disease clinical fellows. Infect Control Hosp Epidemiol 2018;39:1154–1162. 30156171 10.1017/ice.2018.198

[18] Gross R , Morgan AS , Kinky DE , Weiner M , Gibson GA , Fishman NO. Impact of a hospital-based antimicrobial management program on clinical and economic outcomes. Clin Infect Dis 2001;33:289–295. 11438891 10.1086/321880

