# Supplementary figures and images for: A comparison of active versus passive methods of responding to rapid diagnostic blood culture results
Source: Antimicrob Steward Healthc Epidemiol. 2022 May 2;2(1):e75. doi: 10.1017/ash.2022.26 (PMC9726544; doi:10.1017/ash.2022.26)

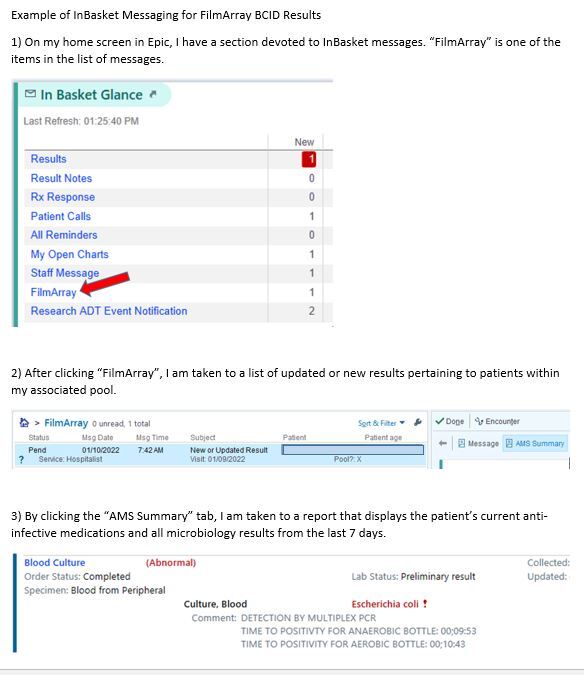

Supplement: Supplementary file 1 [file S2732494X22000262sup001.tiff]
